# Neuroimmunology: What Role for Autoimmunity, Neuroinflammation, and Small Fiber Neuropathy in Fibromyalgia, Chronic Fatigue Syndrome, and Adverse Events after Human Papillomavirus Vaccination?

**DOI:** 10.3390/ijms20205164

**Published:** 2019-10-18

**Authors:** Varvara A. Ryabkova, Leonid P. Churilov, Yehuda Shoenfeld

**Affiliations:** 1 Laboratory of the Mosaic of Autoimmunity, Saint Petersburg State University, Saint-Petersburg 199034, Russian Federation; varvara-ryabkova@yandex.ru (V.A.R.); elpach@mail.ru (L.P.C.); 2 Saint Petersburg Research Institute of Phthisiopulmonology; Saint-Petersburg 191036, Russian Federation; 3 Zabludowicz Center for Autoimmune Diseases, Sheba Medical Center, affiliated to Tel-Aviv University School of Medicine, Tel-Hashomer 52621, Israel

**Keywords:** fibromyalgia, autoimmunity, autoantibodies, neuroinflammation, small fiber neuropathy, chronic fatigue syndrome, HPV vaccine

## Abstract

Fibromyalgia is a disorder characterized by chronic widespread pain and non-pain symptoms, such as fatigue, dysautonomia, and cognitive and sleep disturbances. Its pathogenesis and treatment continue to be the subject of debate. We highlight the role of three mechanisms—autoimmunity, neuroinflammation, and small fiber neuropathy—in the pathogenesis of the disease. These mechanisms are shown to be closely interlinked (also on a molecular level), and the review considers the implementation of this relationship in the search for therapeutic options. We also pay attention to chronic fatigue syndrome, which overlaps with fibromyalgia, and propose a concept of “autoimmune hypothalamopathy” for its pathogenesis. Finally, we analyze the molecular mechanisms underlying the neuroinflammatory background in the development of adverse events following HPV vaccination and suggesting neuroinflammation, which could exacerbate the development of symptoms following HPV vaccination (though this is hotly debated), as a model for fibromyalgia pathogenesis.

## 1. Introduction

Fibromyalgia (FM) is recognized by the World Health Organization under ICD-10 code M79.7 and defined as a chronic widespread pain condition associated with fatigue, sleep and cognitive disturbances, and a variety of somatic symptoms [1,2]. The focus was on the pain for a long time, which could be attributable to the American College of Rheumatology’s (ACR) 1990 classification criteria. These criteria require the existence of chronic widespread pain for more than months, as well as the presence of at least 11 out of 18 specified tender points upon digital palpation [3]. However, FM has a multifaceted nature, as is reflected in the ACR-2010 diagnostic criteria, which changed the definition of FM from a “peripheral pain-defined disease” to a “systemic symptom-based disease” [4]. The ACR-2010 criteria also established a widespread pain index, which replaced the assessment of tender points and required numerical assessment of 41 possible somatic symptoms with individual assessment the severity of 3 major extra-pain symptoms of fatigue, sleep disturbance and cognitive impairments. [2]. The symptoms reported in FM, besides chronic widespread pain and tenderness, include diffuse stiffness, irritable bowel syndrome, fatigue/tiredness, thinking or memory problems, muscle weakness, headache, pain/cramps in the abdomen, numbness/tingling, dizziness, insomnia, depression, pain in the upper abdomen, pelvic pain, vulvodynia, nausea, nervousness, chest pain, blurred vision, fever, dry mouth, itching, wheezing, Raynaud’s phenomenon, hives/welts, ringing in the ears, vomiting, heartburn, oral ulcers, loss of/change in taste, hyposmia, seizures, dry eyes, shortness of breath, loss of appetite, rash, sun sensitivity, hearing difficulties, easy bruising, hair loss, frequent urination, painful urination and bladder spasms, orthostatic intolerance, and temporomandibular joint dysfunction [2,5,6,7]. A modified version of the 2010 criteria has replaced the physician’s estimate of the extent of somatic symptoms with the sum of six self-reported symptoms (fatigue, trouble thinking or remembering, waking up unrefreshed, pain or cramps in the lower abdomen, depression, and headache), making it simpler to use and maintaining sensitivity [8]. The Canadian guidelines for the diagnosis and management of FM syndrome (2012) acknowledge that the criteria for the diagnosis of FM, as developed by the ACR, were primarily intended for research purposes [9]. These guidelines define FM as “a condition that can wax and wane over time and should be diagnosed in an individual with diffuse body pain that has been present for at least three months, and who may also have symptoms of fatigue, sleep disturbance, cognitive changes, mood disorder, and other somatic symptoms to variable degree, and when symptoms cannot be explained by some other illness” [9]. Neither the tender point examination nor any confirmatory laboratory tests are required by this definition. The newest ACTTION-APS taxonomy (AATP) diagnostic criteria for FM (2016) are similar to the Canadian guidelines (2012) with respect to the ease of use in clinical practice. These criteria require only multisite pain (6/9 body areas, present at least three months) and sleep problems OR fatigue, assessed as moderate to severe by the health care professional without any score [10]. These criteria additionally highlight the role of environmental sensitivity in FM by including it in four common features (along with tenderness, dyscognition, and musculoskeletal stiffness), which may be used to support the diagnosis of FM. At the same time, a lack of specific biomarkers for FM diagnosis is a well-known weakness [4], and the elimination of the tender point examination from the modern criteria makes the diagnosis of FM even more subjective.

## 2. Pathogenesis of FM: Autoimmunity

Several features of FM point to an autoimmune component in its pathogenesis. Both trauma and infection, which are capable of triggering autoimmunity, are among the most common events preceding the onset of FM [11]. There are data on the role of various pathogens known to be risk factors for different autoimmune diseases (Epstein‒Barr virus, Herpes simplex virus, hepatitis virus C, *Borrelia burgdorferi*, etc.) in the etiology of FM [11,12,13]. In some cases FM can be temporally related to vaccination, silicone breast implants, or mineral oil injection as part of an autoimmune/inflammatory syndrome induced by adjuvants (ASIA syndrome) [14,15,16]. Like many other autoimmune diseases, FM is characterized by female predominance, which varies due to the different criteria sets and methodology used in epidemiological studies, but also probably due to bias, with a female to male ratio ranging from 1.5:1 to 10:1 [17]. With regard to this gender bias, serum prolactin levels were significantly higher in patients with FM compared to the controls [18]. A significant positive correlation was also observed between prolactin levels and fatigue. The association of FM with B_58_, DR_5_, and DR_8_ HLA alleles supposes probable genetic susceptibility to the autoimmune process [19]. In line with that, a higher prevalence of several autoimmune diseases, including rheumatoid arthritis, systemic lupus erythematosus, ankylosing spondylitis, Sjogren’s syndrome, vasculitis, polymyositis, spondylarthritis, inflammatory bowel diseases, celiac disease, and diabetes mellitus type 1, was found in patients with FM [7]. Activation of the adaptive immune system in FM is supported by the immune cell profile of patients (an increase in all B lymphocytes subsets, CD4^+^CD25^low^ activated T lymphocytes, and CD4^+^HLA-DR^+^ activated T lymphocytes, along with reduced CD4^+^CD25^high^ T lymphocytes and NK cells) [20]. Several autoantibodies (AAb) were found to be elevated in the sera of patients suffering from FM, including those towards 5-hydroxytryptamine, gangliosides, and phospholipids [21,22]. In a cohort of 20 female patients with primary fibromyalgia syndrome, 55% had anti-smooth muscle AAb and 40% had anti-striated muscle AAb, compared to none of the age-matched healthy women [23]. Current data indicate that FM could accompany subclinical stages of such autoimmune diseases as Hashimoto thyroiditis and Sjögren’s syndrome. Anti-thyroperoxidase (TPO) AAb were positively associated with FM in several studies, even in patients without Hashimoto’s thyroiditis and hypothyroidism [24,25,26]. In the subgroup of patients with FM and sicca syndrome and/or xerostomia, 32% tested positive for Sjögren’s syndrome AAb and 26% tested positive for the novel early Sjögren’s syndrome markers only [27].

## 3. Pathogenesis of FM: Neuroinflammation

**Figure 1:**
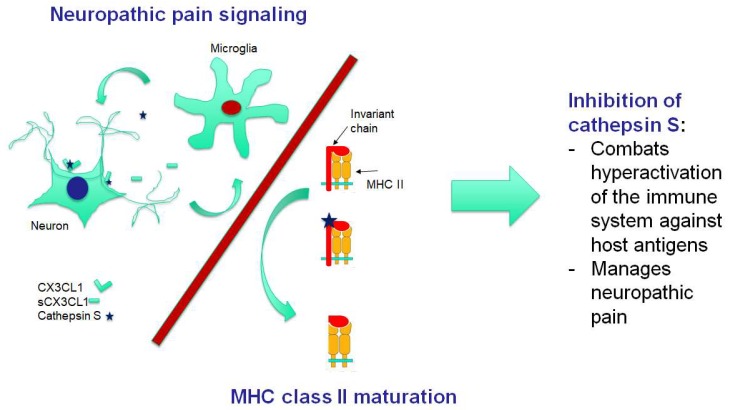
Inhibition of cathepsin S: the mechanism of potential bifunctional therapeutic approach in fibromyalgia. Cathepsin S is involved both in central sensitization, which underlies neuropathic central pain, and in the mediation of the immune response. Activated microglial cells release cathepsin S, which then cleaves CX3CL1 from neurons; this soluble form of the chemokine is active in the stimulation of microglia (positive feedback). Chemokines and cytokines released by activated microglia produce a state of neuronal hyperactivity (central sensitization). In immune cells, cathepsin S cleaves off the invariant chain, which forms a complex with nonmature MHC II molecules in the endoplasmic reticulum and hence blocks the binding of cellular peptides or peptides from the endogenous pathways. Inhibition of cathepsin S blocks the processing of the invariant chain, thereby halting MHC II maturation in the Golgi apparatus before the fusion with a late endosome. Some promising results of cathepsin S inhibition, both in autoimmune diseases and in chronic pain syndrome, are described in the text. The soluble form of CX3CL1 was significantly elevated both in cerebrospinal fluid and the plasma of patients with fibromyalgia.

Neuroinflammation has recently received more attention with respect to FM. It should be mentioned that neuroinflammatory mechanisms are considered to be key links in the pathogenesis of many chronic pain conditions, probably through triggering central sensitization [28]. Analysis of 92 inflammatory-related proteins in cerebrospinal fluid (CFS) and in plasma established evidence for both neuroinflammation and chronic systemic inflammation in FM [28]. One of these proteins, which are significantly elevated both in CFS and plasma, is chemokine CX3CL1 (also known as fractalkine). It is a link to the signaling pathway supposed to be most prominent in experimental models of neuropathic pain [29]. Soluble fractalkine is liberated from primary afferent terminals and surrounding spinal neurons by cathepsin S [30]. Morpholinurea-leucine-homophenylalanine-vinyl sulfone-phenyl (LHVS), an irreversible inhibitor of cathepsin S, was shown to stop the increase of fractalkine [30,31]. Interestingly, LHVS treatment prevents or attenuates experimental autoimmune encephalitis in mice—an animal model of the multiple sclerosis [32]. LHVS or anti-fractalkine antibodies were able to reverse established pain behaviors in rats with collagen-induced arthritis, the animal model of another autoimmune disease (rheumatoid arthritis), but did not slow the development of the clinical signs of the disease [33,34]. With regard to pathophysiological mechanisms, mechanical pressure hypersensitivity and microglial response were significantly attenuated in this model by both LHVS and anti-fractalkine antibodies. Inhibition of fractalkine with anti-fractalkine antibodies improved experimental autoimmune myositis in the mouse model [35]. At the same time, LHVS was shown to produce neuroprotective effects in mice after traumatic brain injury, which often ends in the preservation of symptoms common for FM for several months [36,37]. The mechanism of immunomodulatory effects of LHVS has been established. Cathepsin S controls the proteolysis of the major histocompatibility complex (MHC) II-associated invariant chain, which is a prerequisite for antigenic peptide loading of MHC II. LHVS has been shown to block both the invariant chain processing and antigen presentation in vitro and in vivo by the inhibition of cathepsin S [38,39]. Thus, LHVS may interfere with the activation of self-reactive CD4^+^ T cells by autoantigens [39]. In line with these findings, LHVS caused a dose-dependent reduction of Th1-type (IFNγ), Th2-type (IL-3, IL-5 and IL-13), and Th17-type (IL-17) cytokines [40]. However, the largest response window was obtained with the IFNγ and IL-17 readout, indicating a possible preference for Th1 and Th17 suppression, respectively, by this cathepsin S inhibitor. To summarize, inhibition of cathepsin S combines regulation of both the neuropathic pain and the immune response. This bimodal regulation makes the inhibition of cathepsin S a promising target in the treatment of FM (Figure 1).

There is additional evidence of neuroinflammation in FM. Widespread activation of microglia (most pronounced in the medial and lateral walls of the frontal and parietal lobes) was detected by positron emission tomography in the cortex of patients with FM compared to healthy controls [41]. Higher subjective ratings of fatigue in FM patients were associated with a higher signal in the anterior and posterior middle cingulate cortices. Neuroinflammation in FM was interpreted both as “neurogenic,” presumably triggered by pain and stress [42], and secondary to such concomitant conditions of FM as small intestine bacterial overgrowth (a type of gastrointestinal dysbiosis), vitamin D deficiency, and mitochondrial dysfunction [43]. Notably, all of those concomitant conditions are linked with autoimmunity: small intestine bacterial overgrowth and vitamin D deficiency are considered factors predisposing to some autoimmune diseases [44], and mitochondrial dysfunction is discussed as the consequence of these diseases. In particular, small intestine bacterial overgrowth was associated with inflammatory bowel disease, Sjogren’s syndrome, celiac disease resistant to a gluten-free diet, and seropositivity for anti-TPO AAb [45,46,47,48]. Vitamin D was shown to modulate multiple sclerosis, systemic sclerosis, autoimmune thyroid diseases, rheumatoid arthritis, and primary biliary cirrhosis [49]. Localized or global mitochondrial dysfunction is currently considered an invariant feature of autoimmune diseases and was reported in multiple sclerosis, systemic lupus erythematosus, Sjogren’s syndrome, and rheumatoid arthritis [50]. However, irrespective of the origin of neuroinflammation in FM, it “opens the gate” for immune cells and AAb to the brain [51]. In this case, one could suggest the pathophysiological relevance of AAb to 5-hydroxytryptamine in FM (see above), which is characterized by low-serum serotonin levels and a constellation of symptoms, typical for serotoninergic abnormalities [52]. The same suggestion could be made concerning anti-TPO in FM. This assumption is supported by the association between the presence of diagnosis of mood or anxiety disorder and anti-TPO seropositivity in a general population [53].

## 4. Pathogenesis of FM: Small Fiber Neuropathy

Small fiber neuropathy (SFN) is a structural abnormality of small nerve fibers with the degeneration of the distal terminals of nerve endings [54]. SFN was diagnosed in a major subset of patients with FM by different methods, including the most practical and accurate [55]—skin biopsy with the assessment of the intraepidermal nerve fiber density (IENFD) [56,57,58,59,60,61,62]. IENFD lower than 5 percentiles, which leads to a diagnosis of SFN, was detected in 30‒76% patients with FM [58,59,60,61]. While the pathogenesis of SFN in FM is not well studied, a significant inverse correlation between IL-2R and IENFD was documented in one study [63]. Some of the small neural fibers belong to the sensor neurons, which are able to produce and secrete into inervated areas neuropeptides with anti-inflammatory activity [64]. Their deficit in IENFD can promote inflammation. Another proof of the autoimmune nature of SFN in FM comes from the results of treatment: Intravenous polyclonal immunoglobulin has been proven to be very effective for improving SFN in patients with FM [65]. There are two hypotheses on the relationship between SFN, the immune system, and central sensitization in FM. According to the first one, SFN, most probably immune-mediated, is the peripheral trigger of central sensitization and therefore reinforces pain [57]. The second states that SFN is a result of neuroinflammation in the central nervous system. The sustained increase in insular glutamate triggers SFN in an animal model [54]. Glutamate increase in the brain is a typical feature of neuroinflammation [66], and elevated insular glutamate, associated with experimental pain, was detected in patients with FM [67]. To summarize, SFN is the third important aspect of FM pathogenesis. The relationship between SFN and neuroinflammation appears to be complex. At the same time, it is highly probable that SFN is of an autoimmune nature in FM.

## 5. Chronic Fatigue Syndrome and FM

**Figure 2:**
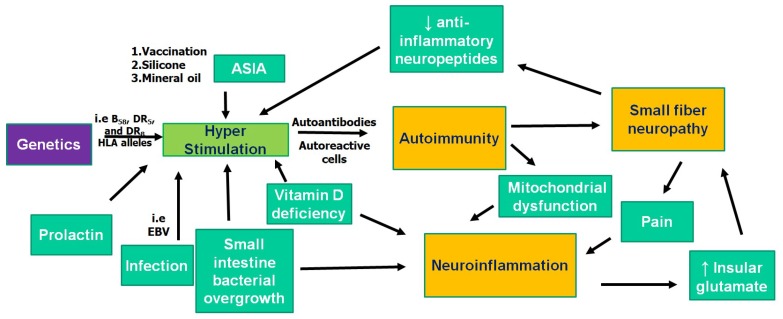
The relationship between three major links of the pathogenesis of FM. The hyperstimulation of the immune system, in addition to genetic predisposition, increases the risk of breach of self-tolerance. Neuroinflammation in FM was interpreted both as “neurogenic,” presumably triggered by pain and stress, and secondary to such concomitant conditions of FM as small intestine bacterial overgrowth, vitamin D deficiency, and mitochondrial dysfunction, which are also linked with autoimmunity. At the same time, there is a two-way relationship between autoimmunity and SFN, as well as between SFN and neuroinflammation (see the text). ASIA: autoimmune/inflammatory syndrome induced by adjuvants, EBV: Epstein-Barr virus, FM: fibromyalgia, SFN: small fiber neuropathy, ↑ increased production, ↓ decreased production.

Chronic fatigue syndrome/myalgic encephalomyelitis (CFS/ME) (ICD-10-CM R53.82 or G93.3 if postviral) is a heterogeneous disease that presents with pronounced disabling fatigue without relief after rest, sleep disturbances, and cognitive impairment [1,68]. The complexity of this disorder is apparent from its autonomic, neuroendocrine and immune manifestations. Substantial evidence for the role of autoimmunity in CFS has been reported recently. These pieces of evidence include: (1) genetics related to alterations of the immune system; (2) association of the onset of the disease with the risk factors common for autoimmune diseases; (3) alteration of immune cells’ subsets; (4) comorbidity with other autoimmune diseases; (5) the production of AAb [69,70,71,72]. Among the different AAb types, a group of AAb against G protein coupled receptors has attracted the most attention. Higher AAb levels against M1, M3, and M4 acetylcholine receptor (AChR) and β2 adrenergic receptor (AdR) were found in CFS patients compared to controls [73]. Their pathophysiological relevance is supported by clinical evidence, including the removal of anti-β2 AdR and anti-M3/M4 AChR AAb in CFS and rapid symptom improvement following immunoadsorption [74]. Sharing many traits with FM, CFS differs from it in terms of the predominant fatigue and postexertional malaise. The evidence for the dysfunction of the hypothalamic‒pituitary‒adrenal axis in CFS is substantial, while the data on its dysfunction in FM are controversial [75,76]. Immune-inflammatory pathways were shown to potentially underpin the hypofunction of the HPA axis in CFS [77]. Neuroinflammation is a common trait of both CFS and FM. The activation of microglia in CFS was observed by positron emission tomography and the signals in the amygdala, thalamus, and midbrain positively correlated with the cognitive impairment score: in the cingulate cortex and thalamus, positively with pain score; and in the hippocampus with depression score [78]. To our knowledge, CFS was first hypothesized to be an autoimmune chronic hypothalamitis by Zaichik and Churilov [79]. An animal model of CFS created by immunization with synthetic analogues of viral polyribonucleotides demonstrated signs of neuroinflammation, glial activation, and serotonin reuptake transporter failure [80]. Development of impairments to the function of the hypothalamic‒pituitary‒adrenal system was also reported in this model. In particular, a decrease in the adrenocorticotropic hormone sensitivity of adrenal cells and suppression of the negative feedback mechanism were detected [81]. Although some researchers did not find any differences between the levels of antineuronal AAb in CFS and healthy individuals [82], and others even found a decrease of AAb towards glial fibrillar acid protein in the sera of patients with CFS, which was correlated with exacerbations of the disease and the presence of Epstein‒Barr virus [83]. While both mast cells [84] and the innate immune system [85] were regarded as triggers for the focal inflammation in the hypothalamus in CFS, the role of the adaptive immune system should also be considered. AdR and muscarinic AChR are expressed in the hypothalamus and regulate the activity of HPA axis and sympathoadrenal system [86,87,88,89,90]. The concept of “autoimmune hypothalamopathy,” which results from the functional effects of anti-G protein coupled receptors AAb on the AdR and muscarinic AChR, appears to be reasonable in CFS. The ability of serum AAb against the muscarinic AChR to affect the brain cholinergic system has been proven with positron emission tomography [91]. The relationship between three major links of FM pathogenesis (autoimmunity, neuroinflammation, and small fiber neuropathy) is summarized in Figure 2. These pathological mechanisms also appear to be involved in the pathogenesis of CFS, which has a lot in common with FM. However, we postulate that the former is characterized by the specific involvement of the hypothalamus (“autoimmune hypothalamopathy” or “hypothalamitis”).

## 6. Human Papillomavirus Vaccination and FM

Vaccination is a great achievement of the public health system. However, vaccines may have side effects, similar to any other therapeutic agent. Like the side effects of other medicines, vaccine side effects most probably manifest in subjects with a genetic predisposition. The side effects reported to develop following human papillomavirus (HPV) vaccination include headache, general fatigue, orthostatic intolerance, dizziness, gait disturbance, sleep disorders, a decreased ability to learn, amnesia, dysphagia, aphasia, hyperventilation, coldness of the legs, limb pain, limb weakness, tremors, pyrexia, myalgia, myositis or muscle weakness, arthralgia and/or arthritis, gastrointestinal dysmotility, and disturbed menstruation [92,93,94,95,96]. While it is difficult to categorize this condition within a specific diagnosis, in a recent study, based on the ACR-2010 questionnaire, which enabled the diagnosis of FM and established its severity, 53% of individuals who reported a chronic disease after vaccination against HPV met the diagnostic criteria for FM [97]. It is important to indicate that, as a reaction to independent publications of case series with similar symptoms following HPV vaccination by authors from Denmark [94] and Japan [95], international health authorities provided several studies on the issue. The European Medicines Agency judged that there was no relationship between the vaccine against HPV and the development of complex regional pain syndrome or postural orthostatic tachycardia [98]. Reviews conducted by British, [99] Canadian [100], and Spanish [101] health authorities supported the safety of immunization against HPV. A powerful argument put forward by the defenders of this vaccine was that the large, double-blind, randomized preclinical studies guaranteed the safety of the HPV vaccine [102]. These randomized studies have a higher level of reliability in evidence-based scientific medicine and eliminate cases that are not related to vaccination itself. The results are totally independent of the judgment of the researchers. However, a critical review of randomized trials and postmarketing case series has also been published [103]. It was suggested by Martínez-Lavín [102] that if the veracity of the complex of symptoms following HPV vaccination in genetically prone individuals is corroborated, it could become a model of FM pathogenesis. The author speculated that SFN and dysautonomia could be the key mechanisms underlying both these conditions. There are grounds for this conclusion. (1) In two different studies, SFN was detected in 17/40 and 20/40 patients with neurologic symptoms following HPV vaccination [95,96]. (2) In those patients who met the criteria for FM, there was a correlation between the severity of FM, as measured by ACR-2010, and the intensity of dysautonomia scored by the composite autonomic symptom score (COMPASS-31). Dysautonomia following HPV vaccination could be a result of the autoimmune process. AAb against different G-protein coupled receptors was looked for in the sera of adolescent girls (*n* = 55) with symptoms of prolonged general fatigue, orthostatic intolerance, chronic regional pain syndrome, and cognitive dysfunction following HPV vaccination [92]. The serum levels of AAb against α1 AR, α2 AR, β1 AR, β2 AR, M1 AChR, M2 AChR, M3 AChR, M4 AChR, M5 AChR, and the endothelin receptor were found to be significantly elevated in the vaccinated girls compared with the controls [92].

In our opinion, attention should also be paid to the second aspect of the FM pathogenesis—neuroinflammation. This component complements post-HPV vaccination phenomena as a model of FM pathogenesis. Although acute disseminated encephalomyelitis, myelitis, optic neuritis, multiple sclerosis, and encephalitis were reported following HPV vaccination [104,105,106], no significant association was found between central demyelination, multiple sclerosis, optic neuritis, and HPV vaccination [107]. However, cognitive and neurological symptoms are an inherent part of the adverse events reported following HPV vaccination [92,108,109]. There are data suggesting that autoimmune encephalitis could be underappreciated in reports on post-HPV vaccination phenomena. In one study, 71% of patients with neurological symptoms, which developed following HPV vaccination, demonstrated an autoimmune encephalitis pattern in the ^123^-I-IMP-SPECT study [96]. Some of them had AAb against ganglionic AChR or gangliosides, while about half of the patients responded well to repeating immune adsorption plasmapheresis under steroids and azathioprine. Besides vaccine antigens, which could cause (due to the molecular mimicry phenomenon) the development of AAb targeting the central nervous system, the second component of HPV vaccines—aluminum adjuvants—could also induce a neuroinflammatory background in patients who develop adverse events following HPV vaccination. Signs of an inflammatory process in the CNS and the toxicity of Al adjuvant/Al-containing vaccines has been reported in different countries and in both mouse and large animal (sheep) models [110]. As was shown, Al particles are able to disseminate from the injection site within immune cells to the lymph nodes and to the brain, from which they do not recirculate [111]. The molecular mechanism of neuroinflammation caused by Al hydroxide particles involves the activation of the NALP3 inflammasome [112]. This is a multimeric protein complex that initiates an inflammatory form of cell death and triggers the release of proinflammatory cytokines IL-1β and IL-18 [113]. In support of this mechanism, IL-1β was detected in both brain immune cells and neurons loaded with Al hydroxide particles in mouse experiments [111]. The effects of the Al adjuvant and the HPV vaccine Gardasil versus the placebo on behavioral parameters in female mice were evaluated [114]. While locomotor activity stayed intact, the results of the forced swimming test indicated the development of depressive behavior in mice injected with Al and Gardasil. Microglial activation in the CA1 area of the hippocampus of Gardasil-injected mice was revealed. Moreover, anti-HPV antibodies from the sera of Gardasil injected mice showed cross-reactivity with the mouse brain protein extract, which could serve as further evidence for the role of the molecular mimicry phenomenon in the development of the described complex of symptoms following HPV vaccination. In view of the preceding, we support the recent concept of considering (the hotly-debated) post-HPV vaccination syndrome as a model for FM pathogenesis [102]. Neuroinflammation could be an underappreciated component common to both these syndromes. Both vaccine antigens (due to the molecular mimicry phenomenon) and aluminum adjuvants (due to potential dissemination in the brain and inflammatory effects) in the HPV vaccines could be involved in the development of neuroinflammation underlying the reported complex of symptoms. This fact should be taken into account when randomized control trials with the HPV vaccine are analyzed, since most of them utilize as a control not a true placebo, but an aluminum adjuvant [102].

## 7. Conclusions

Nowadays, the existence of FM is not in question. However, insight into its pathogenesis, and therefore the possible treatments, remains limited. At the same time, modern diagnostic criteria emphasize that FM is not just chronic widespread pain, but a multisystem entity involving some pathogenesis and a constellation of neurological symptoms, including cognitive, autonomic, and sensory disturbances. There is sufficient evidence that autoimmune factors play a major role in the development of FM. These include genetic predisposition to autoimmune processes, an association with several autoimmune conditions, and a high prevalence of several AAb and immune cell subsets’ alterations. We summarize the relationship between three major links of FM pathogenesis (autoimmunity, neuroinflammation, and small fiber neuropathy) in this paper. One interesting implication of this relationship is the connection between the signaling pathways of neuropathic pain and the triggering of autoimmunity. In this article, we speculate on the possible implementation of this connection in the treatment of FM, highlighting the example of the inhibition of cathepsin S or fractalkine.

## Abbreviation

AAb	autoantibodies
AChR	acetylcholine receptor
ACR	American College of Rheumatology
AdR	adrenergic receptor
CFS	chronic fatigue syndrome
CSF	cerebrospinal fluid
FM	fibromyalgia
HPA	hypothalamic-pituitary-adrenal
HPV	human papillomavirus
IENFD	intraepidermal nerve fiber density
LHVS	morpholinurea-leucine-homophenylalanine-vinyl sulfone-phenyl
MHC	major histocompatibility complex
SFN	small fiber neuropathy
TPO	thyroperoxidase

## References

[1] Boerma T., Harrison J., Jakob R., Mathers C., Schmider A., Weber S. (2016). Revising the ICD: Explaining the WHO approach. Lancet.

[2] Wolfe F., Clauw D.J., Fitzcharles M.-A., Goldenberg D.L., Katz R.S., Mease P., Russell A.S., Russell I.J., Winfield J.B., Yunus M.B. (2010). The American College of Rheumatology Preliminary Diagnostic Criteria for Fibromyalgia and Measurement of Symptom Severity. Arthritis Rheum..

[3] Wolfe F., Smythe H.A., Yunus M.B., Bennett R.M., Bombardier C., Goldenberg D.L., Tugwell P., Campbell S.M., Abeles M., Clark P. (1990). The American College of Rheumatology 1990 Criteria for the Classification of Fibromyalgia. Report of the Multicenter Criteria Committee. Arthritis Rheum..

[4] Sarzi-Puttini P., Atzeni F., Masala I.F., Salaffi F., Chapman J., Choy E. (2018). Are the ACR 2010 diagnostic criteria for fibromyalgia better than the 1990 criteria?. Autoimmun. Rev..

[5] Jones K.D., Hoffman J.H. (2009). Fibromyalgia.

[6] Amital H., Agmon-Levin N., Shoenfeld N., Arnson Y., Amital D., Langevitz P., Gurman A.B., Shoenfeld Y. (2014). Olfactory impairment in patients with the fibromyalgia syndrome and systemic sclerosis. Immunol. Res..

[7] Lichtenstein A., Tiosano S., Amital H. (2018). The complexities of fibromyalgia and its comorbidities. Curr. Opin. Rheumatol..

[8] Wolfe F., Clauw D.J., Fitzcharles M.-A., Goldenberg D.L., Häuser W., Katz R.S., Mease P., Russell A.S., Russell I.J., Winfield J.B. (2011). Fibromyalgia Criteria and Severity Scales for Clinical and Epidemiological Studies: A Modification of the ACR Preliminary Diagnostic Criteria for Fibromyalgia. J. Rheumatol..

[9] Fitzcharles M.-A., Ste-Marie P.A., Goldenberg D.L., Pereira J.X., Abbey S., Choinière M. (2013). 2012 Canadian Guidelines for the Diagnosis and Management of Fibromyalgia Syndrome: Executive Summary. Pain Res. Manag..

[10] Arnold L.M., Bennett R.M., Crofford L.J., Dean L.E., Clauw D.J., Goldenberg D.L. (2019). AAPT Diagnostic Criteria for Fibromyalgia. J. Pain.

[11] Buskila D., Atzeni F., Sarzi-Puttini P. (2008). Etiology of fibromyalgia: The possible role of infection and vaccination. Autoimmun. Rev..

[12] Pridgen W.L., Duffy C., Gendreau J.F., Gendreau R.M. (2017). A famciclovir + celecoxib combination treatment is safe and efficacious in the treatment of fibromyalgia. J. Pain Res..

[13] Reshkova V., Kalinova D., Milanov I. (2015). Evaluation of antiviral antibodies against Epstein-Barr Virus and neurotransmitters in patients with fibromyalgia. J. Neurol Neurosci..

[14] Vera-Lastra O., Hernadez P.B., Sánchez-Rodríguez A., Jara L. (2017). AB0991 Prevalence of fibromyalgia and depression in patients with autoimmune /inflammatory syndrome induced by adjuvants compared to patients with systemic sclerosis. Annu. Eur. Congr. Rheumatol..

[15] Khoo T., Proudman S., Limaye V. (2019). Silicone breast implants and depression, fibromyalgia and chronic fatigue syndrome in a rheumatology clinic population. Clin. Rheumatol..

[16] Agmon-Levin N., Zafrir Y., Kivity S., Balofsky A., Amital H., Shoenfeld Y. (2014). Chronic fatigue syndrome and fibromyalgia following immunization with the hepatitis B vaccine: Another angle of the ‘autoimmune (auto-inflammatory) syndrome induced by adjuvants’ (ASIA). Immunol. Res..

[17] Wolfe F., Walitt B., Perrot S., Rasker J.J., Häuser W. (2018). Fibromyalgia diagnosis and biased assessment: Sex, prevalence and bias. PLoS ONE.

[18] Kilic Baygutalp N., Seferoglu B., Baygutalp F., Senel K. (2013). The correlation of serum prolactin levels and clinical parameters in the patients with fibromyalgia syndrome. Fiz. Tip. Rehabil. Bilim. Derg..

[19] Branco J.C., Tavares V., Abreu I., Correia M.M., Caetano J.A.M. (1996). HLA Studies in Fibromyalgia. J. Musculoskelet. Pain.

[20] Carvalho L.S.C., Correa H., Silva G.C., Campos F.S., Baião F.R., Ribeiro L.S., Faria A.M., Reis D.D. (2008). May genetic factors in fibromyalgia help to identify patients with differentially altered frequencies of immune cells?. Clin. Exp. Immunol..

[21] Klein R., Berg P.A. (1995). High incidence of antibodies to 5-hydroxytryptamine, gangliosides and phospholipids in patients with chronic fatigue and fibromyalgia syndrome and their relatives: Evidence for a clinical entity of both disorders. Eur. J. Med. Res..

[22] Dadabhoy D., Crofford L.J., Spaeth M., Russell I.J., Clauw D.J. (2008). Biology and therapy of fibromyalgia. Evidence-based biomarkers for fibromyalgia syndrome. Arthritis Res. Ther..

[23] Jacobsen S., Wiik A., Høyer-Madsen M., Danneskiold-Samsøe B., Høyer-Madsen M., Danneskiold-Samsøe B. (1990). Screening for autoantibodies in patients with primary fibromyalgia syndrome and a matched control group. APMIS.

[24] Ribeiro L.S., Proietti F.A. (2004). Interrelations between fibromyalgia, thyroid autoantibodies, and depression. J. Rheumatol..

[25] Suk J., Lee J., Kim J. (2012). Association between Thyroid Autoimmunity and Fibromyalgia. Exp. Clin. Endocrinol. Diabetes.

[26] Ekinci B., Uzkeser H., Sevimli H., Macit P.M., Haliloglu S., Carlioglu A. (2017). Fibromyalgia in patients with thyroid autoimmunity: Prevalence and relationship with disease activity. Clin. Rheumatol..

[27] Applbaum E., Lichtbroun A. (2019). Novel Sjögren’s autoantibodies found in fibromyalgia patients with sicca and/or xerostomia. Autoimmun. Rev..

[28] Bäckryd E., Tanum L., Lind A.-L., Larsson A., Gordh T. (2017). Evidence of both systemic inflammation and neuroinflammation in fibromyalgia patients, as assessed by a multiplex protein panel applied to the cerebrospinal fluid and to plasma. J. Pain Res..

[29] Clark A.K., Yip P.K., Grist J., Gentry C., Staniland A.A., Marchand F., Dehvari M., Wotherspoon G., Winter J., Ullah J. (2007). Inhibition of spinal microglial cathepsin S for the reversal of neuropathic pain. Proc. Natl. Acad. Sci. USA.

[30] Flight M.H. (2007). CatS relief. Nat. Rev. Drug Discov..

[31] Clark A.K., Yip P.K., Malcangio M. (2009). The liberation of fractalkine in the dorsal horn requires microglial cathepsin S. J. Neurosci..

[32] Allan E.R.O., Yates R.M. (2015). Redundancy between Cysteine Cathepsins in Murine Experimental Autoimmune Encephalomyelitis. PLoS ONE.

[33] Clark A.K., Grist J., Al-Kashi A., Perretti M., Malcangio M., Al-Kashi A. (2012). Spinal cathepsin S and fractalkine contribute to chronic pain in the collagen-induced arthritis model. Arthritis Rheum..

[34] Nieto F.R., Clark A.K., Grist J., Hathway G.J., Chapman V., Malcangio M. (2016). Neuron-immune mechanisms contribute to pain in early stages of arthritis. J. Neuroinflamm..

[35] Suzuki F., Nanki T., Imai T., Kikuchi H., Hirohata S., Kohsaka H., Miyasaka N. (2005). Inhibition of CX3CL1 (fractalkine) improves experimental autoimmune myositis in SJL/J mice. J. Immunol..

[36] Xu J., Wang H., Ding K., Lu X., Li T., Wang J., Wang C., Wang J. (2013). Inhibition of Cathepsin S Produces Neuroprotective Effects after Traumatic Brain Injury in Mice. Mediat. Inflamm..

[37] Grandhi R., Tavakoli S., Ortega C., Simmonds M.J. (2017). A Review of Chronic Pain and Cognitive, Mood, and Motor Dysfunction Following Mild Traumatic Brain Injury: Complex, Comorbid, and/or Overlapping Conditions?. Brain Sci..

[38] Allen E.M., Vitali N., Underwood S., Sweeney D., Wheeler D., Lawrence C. (2001). Reversible cathepsin S (CATS) inhibitors block invariant chain degradation both in vitro and in vivo. Inflamm. Res..

[39] Fissolo N.M., Kraus M., Reich M., Ayturan M., Overkleeft H., Driessen C., Weissert R. (2008). Dual inhibition of proteasomal and lysosomal proteolysis ameliorates autoimmune central nervous system inflammation. Eur. J. Immunol..

[40] Baugh M., Black D., Westwood P., Kinghorn E., McGregor K., Bruin J., Hamilton W., Dempster M., Claxton C., Cai J. (2011). Therapeutic dosing of an orally active, selective cathepsin S inhibitor suppresses disease in models of autoimmunity. J. Autoimmun..

[41] Albrecht D.S., Forsberg A., Sandström A., Bergan C., Kadetoff D., Protsenko E. (2019). Brain glial activation in fibromyalgia—A multi-site positron emission tomography investigation. Brain Behav. Immun..

[42] Littlejohn G., Guymer E. (2018). Neurogenic inflammation in fibromyalgia. Semin. Immunopathol..

[43] Vasquez A. (2016). Neuroinflammation in fibromyalgia and CRPS is multifactorial. Nat. Rev. Rheumatol..

[44] Perricone C., Shoenfeld Y. (2019). Mosaic of Autoimmunity: The Novel Factors of Autoimmune Diseases.

[45] Polkowska-Pruszyńska B., Gerkowicz A., Szczepanik-Kułak P., Krasowska D. (2019). Small intestinal bacterial overgrowth in systemic sclerosis: A review of the literature. Arch. Dermatol. Res..

[46] Konrad P., Chojnacki J., Kaczka A., Pawłowicz M., Rudnicki C., Chojnacki C. (2018). [Thyroid dysfunction in patients with small intestinal bacterial overgrowth]. Polski Merkur. Lek. Organ. Polskiego Towar. Lek..

[47] Losurdo G., Marra A.M., Shahini E., Girardi B., Giorgio F., Amoruso A., Pisani A., Piscitelli D., Barone M., Principi M. (2017). Small intestinal bacterial overgrowth and celiac disease: A systematic review with pooled-data analysis. Neurogastroenterol. Motil..

[48] Li X., Xiao-Feng L., Gao C., Dong S., Zhang S.-X., Zhao-Hua L. (2019). ab0533 positive rate of small intestinal bacterial overgrowth test (sibo) was significant correlations to disease activity of primary sjogren’s syndrome (pss). Abstr. Accept. Publ..

[49] Rosen Y., Daich J., Soliman I., Brathwaite E., Shoenfeld Y. (2016). Vitamin D and autoimmunity. Scand. J. Rheumatol..

[50] Morris G., Berk M., Walder K., Maes M. (2015). Central pathways causing fatigue in neuro-inflammatory and autoimmune illnesses. BMC Med..

[51] Platt M.P., Agalliu D., Cutforth T. (2017). Hello from the Other Side: How Autoantibodies Circumvent the Blood–Brain Barrier in Autoimmune Encephalitis. Front. Immunol..

[52] Al-Nimer M.S.M., Mohammad T.A.M., Alsakeni R.A. (2018). Serum levels of serotonin as a biomarker of newly diagnosed fibromyalgia in women: Its relation to the platelet indices. J. Res. Med. Sci..

[53] Carta M.G., Loviselli A., Hardoy M.C., Massa S., Cadeddu M., Sardu C., Carpiniello B., Dell’Osso L., Mariotti S. (2004). The link between thyroid autoimmunity (antithyroid peroxidase autoantibodies) with anxiety and mood disorders in the community: A field of interest for public health in the future. BMC Psychiatry.

[54] Harte S.E., Clauw D.J., Hayes J.M., Feldman E.L., St Charles I.C., Watson C.J. (2017). Reduced intraepidermal nerve fiber density after a sustained increase in insular glutamate: A proof-of-concept study examining the pathogenesis of small fiber pathology in fibromyalgia. Pain Rep..

[55] Basantsova N.Y., Starshinova A.A., Dori A., Zinchenko Y.S., Yablonskiy P.K., Shoenfeld Y. (2019). Small-fiber neuropathy definition, diagnosis, and treatment. Neurol. Sci..

[56] Ramírez M., Martínez-Martínez L.-A., Hernández-Quintela E., Velazco-Casapía J., Vargas A., Martínez-Lavín M. (2015). Small fiber neuropathy in women with fibromyalgia. An in vivo assessment using corneal confocal bio-microscopy. Semin. Arthritis Rheum..

[57] Caro X.J., Winter E.F. (2015). The Role and Importance of Small Fiber Neuropathy in Fibromyalgia Pain. Curr. Pain Headache Rep..

[58] Oaklander A.L., Herzog Z.D., Downs H.M., Klein M.M. (2013). Objective evidence that small-fiber polyneuropathy underlies some illnesses currently labeled as fibromyalgia. Pain.

[59] Kosmidis M.L., Koutsogeorgopoulou L., Alexopoulos H., Mamali I., Vlachoyiannopoulos P.G., Voulgarelis M., Moutsopoulos H.M., Tzioufas A.G., Dalakas M.C. (2014). Reduction of Intraepidermal Nerve Fiber Density (IENFD) in the skin biopsies of patients with fibromyalgia: A controlled study. J. Neurol. Sci..

[60] Giannoccaro M.P., Donadio V., Incensi A., Avoni P., Liguori R. (2014). Small nerve fiber involvement in patients referred for fibromyalgia. Muscle Nerve.

[61] de Tommaso M., Nolano M., Iannone F., Vecchio E., Ricci K., Lorenzo M. (2014). Update on laser-evoked potential findings in fibromyalgia patients in light of clinical and skin biopsy features. J. Neurol..

[62] Üçeyler N., Zeller D., Kahn A.-K., Kewenig S., Kittel-Schneider S., Schmid A., Casanova-Molla J., Reiners K., Sommer C. (2013). Small fibre pathology in patients with fibromyalgia syndrome. Brain.

[63] Caro X.J., Winter E.F. (2014). Evidence of Abnormal Epidermal Nerve Fiber Density in Fibromyalgia: Clinical and Immunologic Implications. Arthritis Rheumatol..

[64] Varela N., Chorny A., Gonzalez-Rey E., Delgado M. (2007). Tuning inflammation with anti-inflammatory neuropeptides. Expert Opin. Boil. Ther..

[65] Metyas S., Youssef H., Che C., Quismorio A., Bui J. (2018). Improvement in Fibromyalgia Symptoms and Skin Biopsy Results in Patients with Fibromyalgia and Small Fiber Neuropathy Treated with Intravenous Immune Globulin Infusion( IVIG) [abstract]. Arthritis Rheumatology.

[66] Haroon E., Miller A.H., Sanacora G. (2017). Inflammation, Glutamate, and Glia: A Trio of Trouble in Mood Disorders. Neuropsychopharmacology.

[67] Harris R.E., Sundgren P.C., Craig A., Kirshenbaum E., Sen A., Napadow V., Clauw D.J. (2009). Elevated insular glutamate in fibromyalgia is associated with experimental pain. Arthritis Rheum..

[68] Sharif K., Watad A., Bragazzi N.L., Lichtbroun M., Martini M., Perricone C., Amital H., Shoenfeld Y. (2018). On chronic fatigue syndrome and nosological categories. Clin. Rheumatol..

[69] Sotzny F., Blanco J., Capelli E., Castro-Marrero J., Steiner S., Murovska M., Scheibenbogen C., (Euromene) O.B.O.T.E.N.O.M. (2018). Myalgic Encephalomyelitis/Chronic Fatigue Syndrome—Evidence for an autoimmune disease. Autoimmun. Rev..

[70] Blomberg J., Gottfries C.-G., Elfaitouri A., Rizwan M., Rosén A. (2018). Infection Elicited Autoimmunity and Myalgic Encephalomyelitis/Chronic Fatigue Syndrome: An Explanatory Model. Front. Immunol..

[71] Perez M., Jaundoo R., Hilton K., Del Alamo A., Gemayel K., Klimas N.G., Craddock T.J.A., Nathanson L. (2019). Genetic Predisposition for Immune System, Hormone, and Metabolic Dysfunction in Myalgic Encephalomyelitis/Chronic Fatigue Syndrome: A Pilot Study. Front. Pediatr..

[72] Castro-Marrero J., Faro M., Aliste L., Sáez-Francàs N., Calvo N., Martinez-Martinez A., De Sevilla T.F., Alegre J. (2017). Comorbidity in Chronic Fatigue Syndrome/Myalgic Encephalomyelitis: A Nationwide Population-Based Cohort Study. Psychosomatics.

[73] Loebel M., Grabowski P., Heidecke H., Bauer S., Hanitsch L.G., Wittke K., Meisel C., Reinke P., Volk H.-D., Fluge Ø. (2016). Antibodies to β adrenergic and muscarinic cholinergic receptors in patients with Chronic Fatigue Syndrome. Brain Behav. Immun..

[74] Scheibenbogen C., Loebel M., Freitag H., Krueger A., Bauer S., Antelmann M., Doehner W., Scherbakov N., Heidecke H., Reinke P. (2018). Immunoadsorption to remove β2 adrenergic receptor antibodies in Chronic Fatigue Syndrome CFS/ME. PLoS ONE.

[75] Tomas C., Newton J., Watson S. (2013). A Review of Hypothalamic-Pituitary-Adrenal Axis Function in Chronic Fatigue Syndrome. ISRN Neurosci..

[76] Crofford L.J. (2002). The Hypothalamic-Pituitary-Adrenal Axis in Fibromyalgia: Where Are We in 2001?. J. Musculoskelet. Pain.

[77] Morris G., Anderson G., Maes M. (2017). Hypothalamic-Pituitary-Adrenal Hypofunction in Myalgic Encephalomyelitis (ME)/Chronic Fatigue Syndrome (CFS) as a Consequence of Activated Immune-Inflammatory and Oxidative and Nitrosative Pathways. Mol. Neurobiol..

[78] Nakatomi Y., Mizuno K., Ishii A., Wada Y., Tanaka M., Tazawa S., Onoe K., Fukuda S., Kawabe J., Takahashi K. (2014). Neuroinflammation in Patients with Chronic Fatigue Syndrome/Myalgic Encephalomyelitis: An 11C-(R)-PK11195 PET Study. J. Nucl. Med..

[79] Zaichik A.S., Churilov L. (1999). P Fundamentals of General Pathology. Fundamentals of General Pathophysiology.

[80] Noda M., Ifuku M., Hossain M.S., Katafuchi T. (2018). Glial Activation and Expression of the Serotonin Transporter in Chronic Fatigue Syndrome. Front. Psychol..

[81] Fomicheva E.E., Filatenkova T.A., Rybakina E.G. (2010). Activity in the Hypothalamo-Hypophyseal-Adrenocortical System on Experimental Induction of Chronic Fatigue Syndrome. Neurosci. Behav. Physiol..

[82] Giannoccaro M.P., Cossins J., Sørland K., Fluge Ø., Vincent A. (2019). Searching for Serum Antibodies to Neuronal Proteins in Patients with Myalgic Encephalopathy/Chronic Fatigue Syndrome. Clin. Ther..

[83] Churilov L.P., Danilenko O.V. (2019). [Immunoreactivity in chronic fatigue syndrome during remission, exacerbation and virus carriage] In Russian. Clin. Pathophysiol..

[84] Hatziagelaki E., Adamaki M., Tsilioni I., Dimitriadis G., Theoharides T.C. (2018). Myalgic Encephalomyelitis/Chronic Fatigue Syndrome—Metabolic Disease or Disturbed Homeostasis due to Focal Inflammation in the Hypothalamus?. J. Pharmacol. Exp. Ther..

[85] Mackay A., Tate W.P. (2018). A compromised paraventricular nucleus within a dysfunctional hypothalamus: A novel neuroinflammatory paradigm for ME/CFS. Int. J. Immunopathol. Pharmacol..

[86] Bugajski A., Gadek-Michalska A., Bugajski J. (2006). The involvement of nitric oxide and prostaglandins in the cholinergic stimulation of hypothalamic-pituitary-adrenal response during crowding stress. J. Physiol. Pharmacol. Polish Physiol. Soc..

[87] Smail M.A., Soles J.L., Karwoski T.E., Rubin R.T., Rhodes M.E. (2018). Sexually diergic hypothalamic-pituitary-adrenal axis responses to selective and non-selective muscarinic antagonists prior to cholinergic stimulation by physostigmine in rats. Brain Res. Bull..

[88] Wang D., Feng H., Li Y.-S., Qiu D.-L., Jin H., Jin Q.-H. (2013). β-Adrenoceptors in the hypothalamic paraventricular nucleus modulate the baroreflex in conscious rats. Neurosci. Lett..

[89] Okada S., Yamaguchi N. (2017). Possible role of adrenoceptors in the hypothalamic paraventricular nucleus in corticotropin-releasing factor-induced sympatho-adrenomedullary outflow in rats. Auton. Neurosci..

[90] Hazell G.G.J., Hindmarch C.C., Pope G.R., Roper J.A., Lightman S.L., Murphy D. (2012). G protein-coupled receptors in the hypothalamic paraventricular and supraoptic nuclei—serpentine gateways to neuroendocrine homeostasis. Front. Neuroendocrinol..

[91] Yamamoto S., Ouchi Y., Nakatsuka D., Tahara T., Mizuno K., Tajima S. (2012). Reduction of [11C](+)3-MPB binding in brain of chronic fatigue syndrome with serum autoantibody against muscarinic cholinergic receptor. PLoS ONE.

[92] Ikeda S.-I., Hineno A., Scheibenbogen C., Heidecke H., Shulze-Forster K., Junker J. (2019). Autoantibodies against autonomic nerve receptors in adolescent Japanese girls after immunization with human papillomavirus vaccine. Ann. Arthritis Clin. Rheumatol..

[93] Pellegrino P., Perrone V., Pozzi M., Carnovale C., Perrotta C., Clementi E., Radice S. (2014). The epidemiological profile of ASIA syndrome after HPV vaccination: An evaluation based on the Vaccine Adverse Event Reporting Systems. Immunol. Res..

[94] Brinth L., Theibel A.C., Pors K., Mehlsen J. (2015). Suspected side effects to the quadrivalent human papilloma vaccine. Dan. Med. J..

[95] Kinoshita T., Abe R.-T., Hineno A., Tsunekawa K., Nakane S., Ikeda S.-I. (2014). Peripheral Sympathetic Nerve Dysfunction in Adolescent Japanese Girls Following Immunization with the Human Papillomavirus Vaccine. Intern. Med..

[96] Arata H. (2017). Clinical analysis of neurological symptoms in the patients with HPV vaccination. J. Neurol. Sci..

[97] Martínez-Lavín M., Martínez-Martínez L.-A., Reyes-Loyola P. (2015). HPV vaccination syndrome. A questionnaire-based study. Clin. Rheumatol..

[98] European Medicines Agency Assessment Report EMA/762033/2015. https://www.ema.europa.eu/en/documents/referral/hpv-vaccines-article-20-procedure-assessment-report_en.pdf.

[99] Donegan K., Beau-Lejdstrom R., King B., Seabroke S., Thomson A., Bryan P. (2013). Bivalent human papillomavirus vaccine and the risk of fatigue syndromes in girls in the UK. Vaccine.

[100] Liu X.C., Bell C.A., Simmonds K.A., Svenson L.W., Russell M.L. (2016). Adverse events following HPV vaccination, Alberta 2006–2014. Vaccine.

[101] Rodríguez-Galán M., Pérez-Vilar S., Díez-Domingo J., Tuells J., Gomar-Fayos J., Morales-Olivas F., Pastor-Villalba E. (2014). Notificación de reacciones adversas a la vacuna frente al virus del papiloma humano en la Comunidad Valenciana (2007-2011). Anales Pediatría.

[102] Martínez-Lavín M. (2018). HPV Vaccination Syndrome: A Clinical Mirage, or a New Tragic Fibromyalgia Model. Reumatol. Clín..

[103] Martínez-Lavín M., Amezcua-Guerra L. (2017). Serious adverse events after HPV vaccination: A critical review of randomized trials and post-marketing case series. Clin. Rheumatol..

[104] Sekiguchi K., Yasui N., Kowa H., Kanda F., Toda T. (2016). Two Cases of Acute Disseminated Encephalomyelitis Following Vaccination against Human Papilloma Virus. Intern. Med..

[105] Karussis D., Petrou P. (2014). The spectrum of post-vaccination inflammatory CNS demyelinating syndromes. Autoimmun. Rev..

[106] Hu Y., Tornes L., Lopez-Alberola R. (2018). Two Cases of Pediatric Multiple Sclerosis after Human Papillomavirus Vacciation. Neurology.

[107] Mouchet J., Salvo F., Raschi E., Poluzzi E., Antonazzo I.C., De Ponti F., Bégaud B. (2018). Human papillomavirus vaccine and demyelinating diseases—A systematic review and meta-analysis. Pharmacol. Res..

[108] Blitshteyn S., Brinth L., Hendrickson J.E., Martinez-Lavin M., Blitshetyn S. (2018). Autonomic dysfunction and HPV immunization: An overview. Immunol. Res..

[109] Palmieri B., Poddighe D., Vadalà M., Laurino C., Carnovale C., Clementi E. (2017). Severe somatoform and dysautonomic syndromes after HPV vaccination: Case series and review of literature. Immunol. Res..

[110] Gherardi R.K., Crépeaux G., Authier F.-J. (2019). Myalgia and chronic fatigue syndrome following immunization: Macrophagic myofasciitis and animal studies support linkage to aluminum adjuvant persistency and diffusion in the immune system. Autoimmun. Rev..

[111] Khan Z., Combadière C., Authier F.-J., Itier V., Lux F., Exley C. (2013). Slow CCL2-dependent translocation of biopersistent particles from muscle to brain. BMC Med..

[112] Hornung V., Bauernfeind F., Halle A., Samstad E.O., Kono H., Rock K.L., Fitzgerald K.A., Latz E. (2008). Silica crystals and aluminum salts activate the NALP3 inflammasome through phagosomal destabilization. Nat. Immunol..

[113] Yang Y., Wang H., Kouadir M., Song H., Shi F. (2019). Recent advances in the mechanisms of NLRP3 inflammasome activation and its inhibitors. Cell Death Dis..

[114] Inbar R., Weiss R., Tomljenovic L., Arango M.-T., Deri Y., Shaw C.A. (2017). Behavioral abnormalities in female mice following administration of aluminum adjuvants and the human papillomavirus (HPV) vaccine Gardasil. Immunol Res..

